# Changing the view on spastic movement disorder management to improve active movement competence in the upper motor neuron syndrome: a clinical perspective

**DOI:** 10.3389/fneur.2024.1463292

**Published:** 2024-11-18

**Authors:** Jörg Wissel, Jorge Hernandez Franco

**Affiliations:** ^1^Neurology and Psychosomatic at Wittenbergplatz, Berlin, University of Potsdam, Potsdam, Germany; ^2^Instituto Nacional de Neurología y Neurocirugía Manuel Velasco Suárez, Mexico City, Mexico

**Keywords:** muscle spasticity, stroke, botulinum toxins, flexion synergy, extension synergy, spastic movement disorder, upper motor neuron syndrome, active movement function

## Abstract

It is common in clinical practice to evaluate active movement in spastic movement disorders (SMDs) associated with the upper motor neuron syndrome in terms of resistance to passive movement in the rest position, with the assumption that this may reflect motor control when the patient is in active motion. In addition, the definition of spasticity as a velocity-dependent resistance to passive movement does not account for the impact of abnormal muscle synergies (synkinesia), on active motion of upper and lower limbs in SMDs. In this article, we put forward our theory that synkinetic movement patterns are controlled by activation from spinal afferents and inhibition from the cortex, and become disturbed following a loss of inhibition and change to spinal afferents following damage to the corticospinal tract. In this regard, we propose a change in the focus from passive to active function at the evaluation stage of the SMD management plan, and a new treatment approach to modulate muscle synergies with botulinum neurotoxin type A therapy.

## 1 Introduction

The characterization of spasticity as velocity-dependent muscle resistance to passive movement and a component of the upper motor neuron syndrome (UMNS) has been criticized on the basis that a clinical sign is being described in physiologic terms (1, 2). The European SPASM consortium defined spasticity as involuntary muscle overactivity (3), reflecting the positive features of spasticity in the context of the UMNS, while excluding the negative features, such as paresis and contractures. In contrast, Gracies (4) described the concept of spastic deforming paresis, which includes changes in muscle tissue related to paresis, and different forms of muscle overactivity and antagonistic dyscoordination—the term “spastic myopathy” being used to describe the prolonged shortening of muscles involved. Moreover, the term “spasticity” does not account for the consequences of abnormal muscle synergies in active motion of upper and lower limbs in spastic movement disorders (SMDs) (5) following upper motor neuron (UMN) lesions.

It seems that the term “spasticity” is being accepted as an umbrella term encompassing all kinds of muscle overactivity, and has been used to refer to an etiology at subcortical, brainstem, and spinal level, or to a clinical expression at joint level (6, 7). This may be due to a lack of consensus regarding the terms that might be used during the patient’s evaluation and goal-setting, or clinical evaluation using spasticity scales that measure resistance to passive movement in the rest position (e.g., sitting or supine) with either a defined degree of velocity, including the Ashworth Scale (AS) and modified AS, or varying degrees of velocity, including the Tardieu Scale (TS) and modified TS. Furthermore, there is a tendency in current clinical practice to evaluate active movement in UMNS-associated SMDs in terms of patient-specific active goals (8, 9), rather than measure them as changes in kinesiologic parameters, such as the change in velocity or acceleration in a defined ballistic movement (e.g., elbow extension during reaching movement or knee extension during swing phase of walking), which could offer quantifiable evaluation of SMD in specific everyday active movements of the upper and lower limb.

In this regard, confusion arises from the assumption that a motor disorder, elicited when a person is asked to lie passively, may have an impact on motor control when the patient is actively moving (1). This evades a deeper understanding of impaired motor control (1), and raises the question of whether passive measurement is representative of the problems occurring during active, functional tasks (6).

In addition, spastic co-contraction is absent at rest, by definition, referring to increased antagonist muscle recruitment triggered by the volitional command of agonist muscles in the absence of a phasic stretch, as assessed by electromyograph (10). However, co-contraction may lead to abnormal restricting arm movement patterns, especially in subjects with more severe motor impairment. Although co-contraction is more likely linked to impaired motor function than is spasticity (11), clinicians continue to use passive stretching assessments, inferring velocity dependent increase in muscle tone, as a marker of muscle overactivity during movement. This view has limited the evaluation of other forms of muscle overactivity and the impact of co-contraction on voluntary movement.

Positive signs of the UMNS include stretch-sensitive phenomena (e.g., velocity-dependent increase in muscle tone, clonus, and spasms), spinal phenomena (e.g., disturbed antagonistic muscle activation resulting in increased co-contraction) (8, 12), cortical phenomena (e.g., associated involuntarily increased frequency or magnitude of muscle activity, movement, or movement patterns), and spastic dystonia, which is a spontaneous co-contraction of antagonists, believed to originate from lesions at the cortical level following Denny-Brown’s experiments in 1966 (10). Negative signs consist of loss of strength and dexterity, and impaired motor control and easy fatigability (12); these signs can be described as abnormal, weak activation patterns, or incomplete muscle synergies.

Several studies have investigated the idea that disturbances in muscle synergy, and not muscle spasticity, contribute to dysfunction in active movement in patients with SMD (13–15). The abnormal muscle synergy associated with the UMNS is termed “synkinetic movement” or “synkinesia,” a disorder in which an involuntary movement occurs in combination with a voluntary movement (16). Synkinetic movement is most seen in facial muscles, but has also been described in arm and hand muscles (16, 17).

Spasticity becomes a target for treatment in several conditions, when the focus should be on improving participation and quality of life (1), and reducing the dysfunction from pathologic muscle synergies. Although this article focuses on post-stroke spasticity (PSS), this scenario exists in the treatment of other conditions, including cerebral palsy, multiple sclerosis, and spinal cord injury. There is a need to develop multiparameter methods to broaden the understanding of the neural organization of muscle synergies, and to promote their application in movement analysis and treatment of SMD in rehabilitation (18).

The authors posit that synkinetic movement patterns, which are programmed in hard-wired structures in the spinal cord in the region of the intuminentia cervicalis spinalis and the so-called stepping generator in the lumbal cord, are controlled by activation from spinal afferents and inhibition from supraspinal centers or the cortex. Consequently, when the corticospinal tract (CST) and parapyramidal tracts are damaged, synkinetic movements are disturbed by a loss of inhibition and change in spinal afferents. On this basis, we propose changing the focus of attention from passive function to active function at the evaluation stage of the SMD management plan, and modulating muscle synergies with botulinum neurotoxin type A (BoNT-A) therapy as a new treatment approach.

## 2 Spasticity and spastic movement disorder

A discrepancy has been observed between spasticity due to reflexes in the passive state, and functional SMDs owing to reflexes in the active state (19). The target of most treatment approaches has been to attenuate or abolish reflex activity and reduce velocity-dependent hypertonia of muscle groups, based on the understanding that exaggerated reflexes caused by a loss of inhibition, with resultant overactivity at the spinal level, might be responsible for muscle hypertonia. However, functional movement studies show no relationship between exaggerated reflexes (and their modulation by treatment) and SMD following a spinal or supraspinal lesion, suggesting that reducing clinical signs of velocity-dependent increase in muscle tone, as per Lance’s definition of spasticity (20), does not improve the clinical consequences of movement quality and quantity in SMD (19).

We support the move away from focusing on the abnormalities of the stretch reflex at rest, and towards the interaction of the descending pathways with the spinal circuitry during voluntary movement, which manifests itself in the clinical presentation of SMD.

### 2.1 The descending pathways

The descending spinal tracts or pathways include the CST, reticulospinal tract (RST), rubrospinal tract, tectospinal tract and vestibulospinal tract. Each descending pathway involved in motor control has several anatomic, molecular, pharmacologic, and neuroinformatic characteristics, and functions as part of a large motor network, rather than as separate controllers of the spinal cord (21). A given descending pathway involved in motor control can mediate many different functions (21, 22); for example, the CST mediates fine distal movements, particularly of the hand, while the RST connections are widely assumed to be responsible for coordinated gross movements, primarily of proximal muscles.

The RST, along with the corticoreticular tract (CRT), forms part of the larger corticoreticulospinal tract (CRST) system. The CRST is believed to reorganize after CST injury (e.g., following a stroke) in animals, with limited evidence from human studies (23). It has been suggested that the contra-lesional lateral and medial CRSTs underlie abnormal co-activation of muscles following stroke (13). For example, when damage occurs to the CST and CRST after a subcortical stroke on one hemisphere, the output signal diminishes, causing upregulation of RST excitability in the contra-lesional hemisphere, which appears to be related to abnormal motor synergy and disordered motor control in stroke survivors (7). This suggests a relationship between abnormal muscle synergy and the UMNS, since the same neural circuits, mainly the RST, participate in their development. A unifying account has been proposed to explain the role of RST hyperexcitability in PSS and its related movement impairments, including abnormal muscle synergy and disordered control, in addition to its interactions with motor cortices in motor recovery (7).

## 3 Abnormal muscle synergies

Nonzero simultaneous activation of muscles with opposing actions is referred to as agonist–antagonist co-activation or simply co-activation (24, 25). Co-activation is not limited to muscle pairs; muscle synergy is a consistent pattern of activity across groups of muscles simultaneously involved in the performance of a movement (25, 26). Muscles are co-activated if there is simultaneous excitation of motor neurons of two muscles (26). Conversely, activity in one muscle may coincide with inactivity in another, due to reciprocal inhibition (26), where automatic antagonist alpha motor neuron inhibition is evoked by contraction of the agonist muscle (27).

There is increasing recognition that the major functional deficits following brain damage are largely due to negative features including weakness and loss of dexterity, rather than positive features such as spasticity (28). Abnormal muscle synergies describe the negative signs of the UMNS, where several motor signs are believed to contribute to reduced functional ability: insufficient muscle activation (weakness), and the inability to activate a specific pattern of muscles (reduced selective motor control) or the correct pattern of muscles during movement and/or to accomplish a task (15).

In healthy individuals, activity in the motor cortex, which descends via the CST, is the predominant driver of voluntary movements to the distal upper limb segments (hand and fingers) (21, 26). However, to compensate for stroke-induced damage to the CST, flexor and extensor synergies may emerge due to upregulation of diffusely projecting brainstem motor pathways (26, 29).

In the intact nervous system, there is simultaneous activation of the brainstem pathways driving shoulder muscles with activation of the elbow and hand, due to the nervous system’s diffuse multi-joint projections, which may be utilized for postural adjustments and/or to provide multi-joint stability (29). In addition, the CST and its cortico-reticular projections can selectively “gate” or inhibit any unwanted reticulospinal effects at other joints (29). However, following a stroke there is no suppression of these unwanted effects, resulting in the flexion and extension synergy pattern, the degree of which is determined by the strength of the brainstem pathway (29). Moreover, due to plasticity along the neuroaxis, the contribution of these pathways may come to the fore as new synergies develop at the chronic stage after stroke (26).

Although current muscle synergy models can explain some motor control mechanisms and capture abnormal synergies, the neural implementation of motor control processes by specific central nervous system structures remains largely unknown (18).

### 3.1 Modulating muscle synergies

While only a few spinal circuits have been shown to underlie the abnormalities of patients at rest (30), there is even less known about what is happening during movement. Spasticity management currently focuses on modulation of muscle tone and passive function, and measurement of impairment under passive conditions (13), with the aim of achieving improvement in active function. However, spasticity does not manifest itself when the patient is active in movement, that is, spasticity-related flexor activation has only a small role in motor dysfunction during active movement (13).

A study whereby a robotic device systematically modulated shoulder abduction loading during ballistic movement, concluded that future work should investigate the effectiveness of targeting abnormal flexion synergy, as opposed to flexor spasticity, in the restoration of arm function (13). Moreover, an earlier study demonstrated that specifically targeting flexion synergy using progressive shoulder abduction loading in chronic severe stroke could improve reaching function, despite the persistence of spasticity (14) serving as an example of preserved motor acquisition and motor transfer, despite the presence of impaired motor output following stroke.

## 4 Treating the dysfunction, not the muscle tone

**Figure 1:**
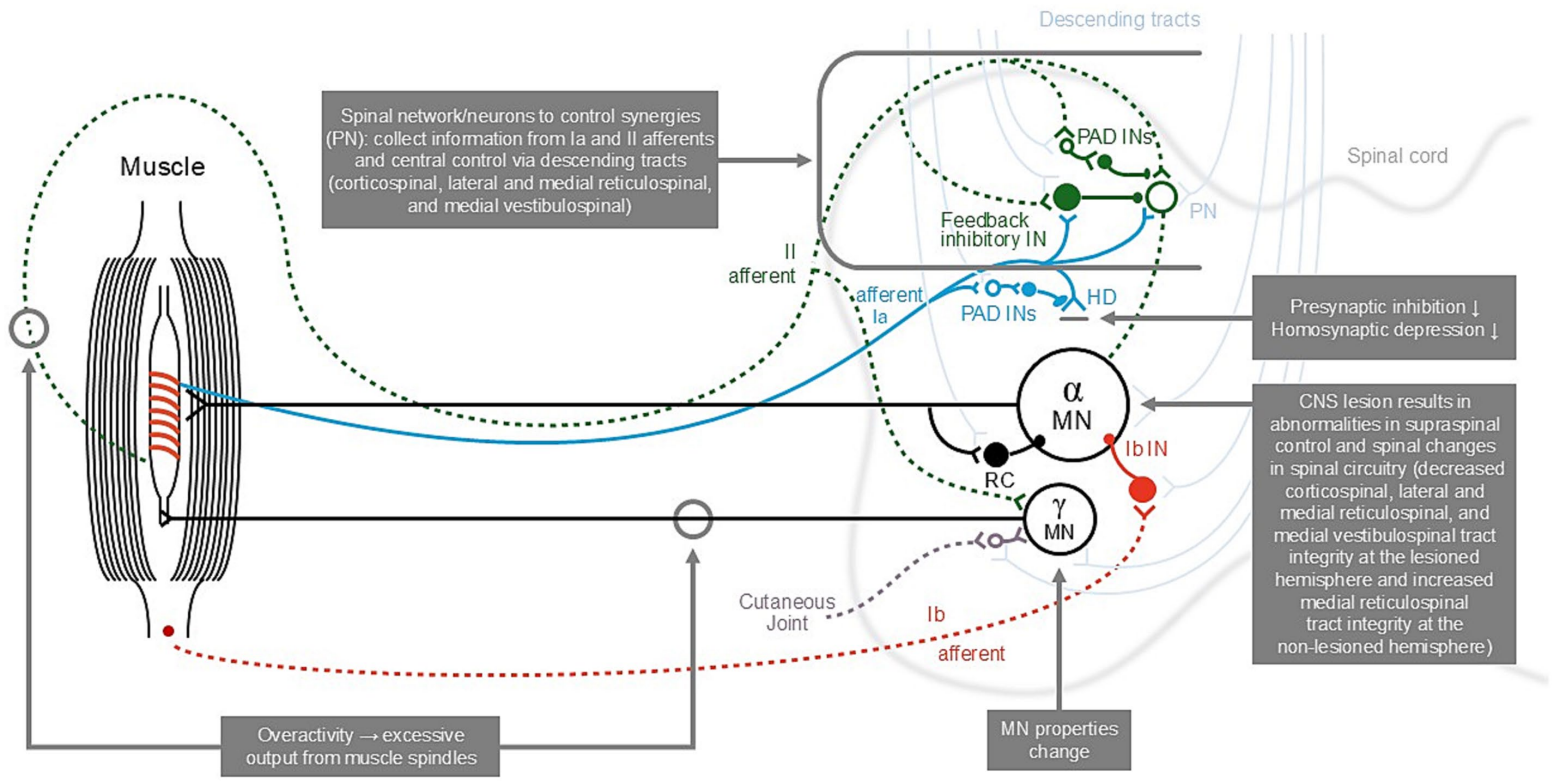
The descending pathways involved in increased muscle tone and the lower-limb reflex [adopted from (30, 44)]. CNS, central nervous system; HD, homosynaptic (“post-activation”) depression; Ia, group Ia afferents from primary spindle endings; Ib, group Ib afferents from Golgi tendon organs; II, group II afferents from secondary spindle endings; IN, interneuron; MN, motoneuron; PAD INs, interneurons mediating presynaptic inhibition; PN, proprioceptive neurons; RC, Renshaw cell responsible for recurrent inhibition.

Due to a lack of appropriate measurement techniques, there is a dearth of information on active function in spasticity. However, it is known that during movement, the intuminentia cervicalis spinalis at the C3–C4 propriospinal system determines activation of the appropriate muscle synergies—an inter-neuronal system, located within the C3–C4 segments, receives input from corticospinal, reticulospinal, and tectospinal systems, as well as feedback from peripheral afferents in the limb (Figure 1) (30). Following subcortical stroke (e.g., capsula interna stroke), there may be a greater transmission of the command for movement through this descending brainstem and peripheral feedback system, and as this system is hard-wired, this may produce unwanted synkinetic movements, adding to or resulting in the classic hemispastic posture or movement pattern (30). In other words, the synkinesia presents as the typical spastic upper limb pattern.

### 4.1 The C3–C4 propriospinal system

The location of the stroke lesion and properties of the secondary descending pathways and their regulation are critical in shaping the synergies in the remaining motor behavior, and consideration of the integrity of remaining descending motor pathways may aid in the design of new rehabilitation therapies (26).

Experiments in cats showed that after lesioning of the CST and rubrospinal tracts at the level of C5, the C3–C4 propriospinal system of spinal cord interneurons played a key role in the control of fine movements and the processes compensating for motor deficiency; after lesioning at the level of C2, however, the leading role was played by ipsilateral tracts in the ventral part of the spinal cord (31).

A study on the C3–C4 segments of macaque monkeys found that di-synaptic inhibitory postsynaptic potentials were induced following the stimulation of the lateral reticular nucleus in some motor neurons (33). A preceding study concluded that tri-synaptic cortico-motoneuronal inhibition occurs following consecutive activation of C3–C4 propriospinal neurons and segmental Ia inhibitory interneurons in the cat (34). In addition, it was demonstrated that these excitatory C3–C4 propriospinal neurons have ascending projection to the lateral reticular nucleus (35). Therefore, it is likely that the di-synaptic inhibitory postsynaptic potentials of the lateral reticular nucleus were mediated by an excitatory C3–C4 propriospinal neurons and Ia inhibitory interneurons (33).

### 4.2 Ia inhibitory interneuron

It is hypothesized that alterations in inhibitory mechanisms in spinal neuronal circuitry are more at play than excitatory processes in a patient with SMD during active movement, although these processes may be inter-related (12). There may be inhibition of the monosynaptic Ia excitation that underlies the dynamic and tonic components of the stretch reflex by spinal reflexes pathways, including presynaptic inhibition of Ia afferent terminals, di-synaptic reciprocal Ia inhibition from muscle spindle Ia afferents from the antagonist muscles, and recurrent inhibition via motor axon collaterals and Renshaw cells (12).

In the absence of damage to the CST, extensor and flexor muscles work in synchrony to control a joint, where activity of the agonist muscle coincides with inactivity of the antagonist muscle through reciprocal inhibition by the Ia inhibitory interneuron (36). In PSS, muscle contraction patterns of the upper limb appear as co-contraction of agonist and antagonist muscles, in addition to co-contraction of other muscles of the upper limb extremity (37), due to the loss of reciprocal inhibition during voluntary command (36).

We propose that reducing the effect of the Ia interneuron will allow the antagonist muscle to work against the spastic agonist muscle, thereby reducing the presenting spastic pattern.

### 4.3 Botulinum neurotoxin type-a treatment

Although neurorehabilitation encompasses both the positive and negative features of the UMNS, BoNT-A therapy addresses the positive features only, where the aim is to decrease the tone in agonist muscles (38). BoNT-A injection may also reduce the spastic co-contraction of the non-injected antagonist muscles by increasing reciprocal inhibition from a more relaxed, and therefore stretched, agonist, or through decreased recurrent inhibition from the injected muscle (39). In other words, there is a benefit to temporarily weakening the muscle with BoNT-A, where injection of the spastic agonist muscle may, in effect, strengthen the antagonist muscle.

This is of interest in neurorehabilitation, as co-contraction is probably a more disabling form of muscle overactivity than spasticity (36, 40). Therefore, it may be beneficial to shift the focus of treatment to the negative signs of the UMNS.

In clinical practice, dynamic evaluation of all muscles should be considered before the patient is treated with BoNT-A. We propose injecting both the agonist and antagonist muscle with BoNT-A, to reduce the stretch reflex of the agonist spastic muscle, while simultaneously reducing the influence of the Ia interneuron on the antagonist muscle. The antagonist should receive a low dose of BoNT-A—enough to block the muscle spindles without inducing functionally relevant paresis. To date, however, there is no conclusive evidence of improvement in active function following BoNT-A injection in spastic co-contraction.

### 4.4 Evidence from clinical practice

In our clinical practice, injecting the biceps brachii (spastic agonist) with BoNT-A, with concurrent injection of the long head of the triceps brachii (antagonist muscle) with a lower dose of BoNT-A, enabled the patient to extend and flex the elbow more smoothly and in a wider range. The combination of injections in the flexors and one extensor of the elbow (thereby blocking the Ia afferents) resulted in weakening of the flexor synergies and better activation of the extensors (medial and lateral head of the triceps) of the elbow; this also improved the active range of movement. These findings show not only a direct effect on spastic muscle (spastic flexor synergy/agonist/flexor) but also a reduction in co-contraction as a result of improved reciprocal inhibition via blocking of Ia afferents to the spinal cord.

Similarly, BoNT-A injection of the gastrocnemius (spastic agonist) resulted in reduced Ia afferents of the tibialis anterior (antagonist muscle), enabling the patient to better activate foot extensors during the swing phase while walking, at 5 weeks post-injection compared with baseline (41). In this case, only the spastic synergy/spastic agonist was injected resulting in the weakening effect of BoNT. However, blockade of the Ia-afferents from the ankle flexors to the spinal cord also occurred, causing disinhibition of the extensors of the ankle (tibialis anterior and peroneii) and thus better extension of the ankle (foot elevation during swing). This is an example of improvement of reciprocal inhibition/improvement in co-contraction during active swing-phase in the gait cycle.

Gracies et al. (42) demonstrated improved active motion and reduced resistance to passive movement in patients with hemiparesis following BoNT-A injection into co-contracting antagonist muscles, and suggested that future policies on BoNT-A therapy be based on evidence for co-contraction impeding active movement, and not only on hypertonia restricting passive movement. They further showed that these effects were observed in specific joints—the finger, wrist and elbow—during active movement (43). The application of these methods could extend beyond injecting muscles involved in antagonistic co-contraction, for example, where the co-contraction of proximal muscles triggered by distal muscle contraction causes disturbance of coordinated movement in a synergistic movement and negatively impacts functional recovery of the upper limb (37).

## 5 Conclusion

Going beyond an agonist–antagonist relationship, regarding spastic paresis as part of a more complex alteration of the sensorimotor network that results in abnormal muscle coordination, and focusing treatment on the SMD-related dysfunction instead of the muscle tone, could open a new area for movement research in patients with SMDs following stroke. Analysis of muscle activity may provide a better understanding of the functional neural deficits in the impaired nervous system and methodology has been published, using visual or computational analyses to extract muscle synergies from poly-electromyographic datasets from affected limbs, to reveal underlying patterns that may reflect different levels of neural function and hence motor function deficiencies (44). Such analyses of muscle activity could occur during standardized motor tasks such as (from simple to complex) grasp and release of a cup, grasp of a cup with water from a table and drink from it, put the cup back on the table and release grip from the cup for the upper limb; and standing, walking, and running for the lower limb. In addition, evaluation of synergy expressions among proximal and distal joints, by using isometric joint torques and surface electromyography, has provided new insights in synergies activity and resulted in suggestions for novel rehabilitation interventions (29). However, evaluation of synergies, as a means of evaluating voluntary movement in post-stroke patients, is still in a developmental phase. To improve movement capacity in patients with the UMNS, future research should center upon multimodal treatments to modify muscle synergies, such as injecting both the agonist and antagonist muscle with BoNT-A (a higher dose in the spastic agonist), in combination with electrical stimulation and active training of the antagonist.

## Data Availability

The original contributions presented in the study are included in the article/supplementary material, further inquiries can be directed to the corresponding authors.
